# Locomotor activity in common spiny mice (*Acomys cahirinuse*): The effect of light and environmental complexity

**DOI:** 10.1186/1472-6785-4-16

**Published:** 2004-11-10

**Authors:** David Eilam

**Affiliations:** 1 Department of Zoology, Tel-Aviv University, Ramat-Aviv 69 978, Israel

## Abstract

**Background:**

Rodents typically avoid illuminated and open areas, favoring dark or sheltered environments for activity. While previous studies focused on the effect of these environmental attributes on the level of activity, the present study tested whether the spatio-temporal structure of activity was also modified in illuminated compared with dark and complex compared with open arenas. For this, we tested common spiny mice (*Acomys cahirinus*) in empty or stone-containing arenas with lights on or lights off.

**Results:**

In an illuminated or open arena, spiny mice moved in less frequent but longer trips with relatively long distances between consecutive stops. In contrast, in either a dark arena or an arena with stones, the animals took shorter and more frequent trips, with more stops per trip and shorter inter-stop distances. In illuminated arenas spiny mice remained mainly along the walls, whereas locomotion in the center was more prevalent in dark empty arenas, and was carried out along convoluted paths. Increasing environmental complexity by adding stones to either illuminated or dark arenas increased locomotion along straight trajectories and away from walls.

**Conclusions:**

Earlier findings of reduced activity in illuminated or open areas have been extended in the present study by demonstrating changes in the spatio-temporal structure of locomotor behavior. In the more complex arenas (with stones) spiny mice traveled along short straight segments whereas in the open their trips were longer and took the shape of a zigzag path which is more effective against fast or nearby predators. Alternatively, the zigzag path may reflect a difficulty in navigation.

## Background

Rodents typically avoid illuminated and open areas, favoring dark or sheltered environments for activity. Indeed, higher activity was described in numerous field and laboratory studies of nocturnal species tested in the dark, compared with their activity when tested under light. For example, common spiny mice (*Acomys cahirinus*) decreased activity and foraging in open spaces under moonlit compared with dark nights [1]. When tested in the dark, laboratory rats increase their activity and display behaviors that indicate reduced habituation, fear and anxiety [2]. Deermice (*Peromyscus maniculatus*) were shown to reduce activity in the open during moonlit nights and were active only near objects such as rocks, grasses, and walls, where they could successfully evade a predator attack [3]. Thus, it appears that rodents perceive increased risk of predation in open spaces and/or during moonlit nights and in consequence shift their activity to more protected microhabitats [4-7]. While many of these studies used indirect measures of locomotor activity, such as footprints [8], the present laboratory study was aimed at direct observation of locomotor behavior under various light levels and arena complexities.

The 'open field' is a widely used apparatus in laboratory studies of rodents' locomotor activity [9]. This apparatus has been criticized for being "a poor and explicitly aversive environment with excess light and open spaces..."[10]. Nevertheless, it is a relatively simple testing environment for a variety of species, in which they display a typical behavioral structure [11] that withstands drastic environmental changes [12]. Studies in wild and laboratory rodents in an illuminated open field (e.g., [9,11-14]) have shown that their locomotor behavior is organized in reference to a key location – the home-base. At the home base, the rodent demonstrates typical behaviors (e.g. grooming and crouching), and sets out on round trips in the area. The building block of the round trip is a stop, with an upper limit of 8–10 stops per trip [15]. The limited number of stops/trip is preserved by scaling the distance between successive stops and adjusting the number of trips, even under large changes in arena size [12,16]. Accordingly, rodents in a larger area made fewer yet longer trips, whereas in a small area they made shorter but more frequent trips. Following these earlier studies, the present study tested open field behavior under varying light level and arena complexity.

The common spiny mouse (*Acomys cahirinus*) was selected for this study since it is a strictly nocturnal species that displaces other species to crepescular or diurnal activity [17,18]. Common spiny mice were thus expected to be sensitive to tests in illuminated compared with dark environments. Also, they live in rocky environments, nimbly foraging in crevices between and under rocks and boulders [18,19], where the complex habitat structure provides shelter and escape from predators. They were thus expected to be also sensitive to changes in environmental complexity. Three questions were posed in this study of common spiny mice: i) is their behavior in an illuminated arena similar to that seen in mice, rats, and voles? ii) does behavior change in dark arena and/or with increased environmental complexity? iii) what is the functional mechanism that may underlie behavioral changes in dark or complex environments? As shown below, activity increased in dark or in complex environment, and took a different form of short straight trajectories. In contrast, spiny mice traveled through the center in a convoluted path in either lit or dark empty arenas. This later form of progression may have a defensive advantage.

## Results

### Level of locomotor activity

**Figure 1:**
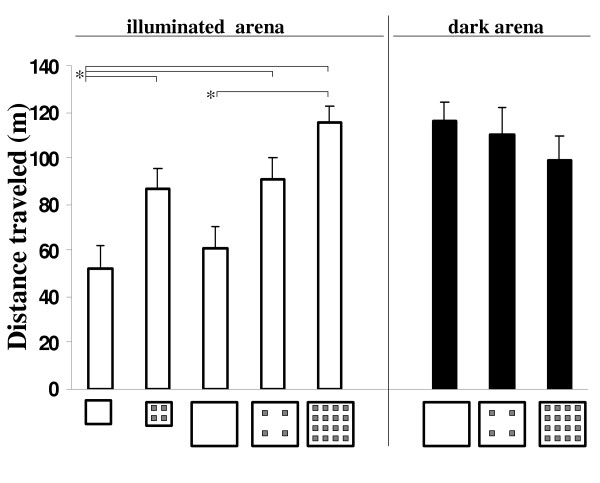
Distance traveled (mean ± SEM) in the lit arenas (open bars) and dark arenas (dark bars). Arena size and number of stones in each arena are depicted along the x-axis. Significant comparisons, as revealed in Tukey HSD test, are depicted by lines at top left. As shown, traveled distance did not change with only arena size. Adding four stones to a small arena significantely increased traveled distance (compare small arena with 0 and with 4 stones); however, adding 4 stones to a large arena did not have a significant effect. In the dark arena, the number of stones did not have a significant effect on the traveld distance.

In the illuminated arena, the distance traveled by spiny mice was significantly affected by the density of stones. In contrast, neither the number of stones nor arena size alone significantly increased activity. As shown for small arena in Fig. 1, traveled distance was significantly greater when four stones were present than when stones were absent. Traveled distance was not greater in comparing small with large empty arenas, or small with large 4 stone arenas. Therefore, changing arena size alone did not increase activity. However, stone density of four/m2 significantely increased activity, as shown for small arena with four stones or large arena with 16 stones (Fig 1). This trend of increased activity with increased stone-density was also echoed in traveling speed (Table 1).

**Table 1:** Parameters of locomotion in an illuminated small arena (1 × 1 m) and large arena (2 × 2 m). For most variables, values increased when stones were added or when arena size was increased. Bonferroni adjustment of p-value was calculated by P = 0.05/10 = 0.005. Mean (± SEM) are followed by superscript numerals that indicate the significantely different test groups (as appeared in the top row) in Tukey Honest Significant Difference (HSD) test.

	Small arena		Large arena			
			
	Empty (1)	4 Stones (2)	Empty (3)	4 Stones (4)	16 Stones (5)	F_45_;p
**Level of activity**						
Traveled distance (m.)	52.0 ± 9.7 ^2,4,5^	86.7 ± 8.8^1^	60.9 ± 9.5^5^	90.4 ± 9.7^1^	115.6 ± 7.3^1,3^	**8.68; <0.0001**
Speed (m/sec)	0.10 ± 0.02^2,3,5^	0.15 ± 0.04^1,5^	0.12 ± 0.02^4,5^	0.18 ± 0.02^1,3,5^	0.36 ± 0.12^1–4^	**5.68; = 0.0012**
**Temporal Structure**						
Stops	87.0 ± 31.4^2,4,5^	228.6 ± 55.7^1,3,4^	51.2 ± 13.9^2,4,5^	146.9 ± 23.9 ^1–5^	235.8 ± 48.6 ^1,3,4^	**7.15; = 0.0002**
# of trips	18.5 ± 5.5^2,4,5^	41.4 ± 11.0^1,3^	12.2 ± 5.0^2,4,5^	38.5 ± 7.8^1,2,5^	74.7 ± 25.2^1,3,4^	**5.81; = 0.001**
Stops/trip	5.0 ± 0.6	5.6 ± 0.4	5.5 ± 1.2	4.4 ± 0.6	3.6 ± 0.3	0.95; ns
Trip length	4.9 ± 1.4^3,5^	2.4 ± 0.4^3^	10.2 ± 2.3^1,2,4,5^	3.1 ± 0.6^3,5^	2.0 ± 0.3^1,3,4^	**5.88; = 0.0009**
Inter-stop distance (m.)	0.94 ± 0.22	0.43 ± 0.06	2.68 ± .1.14	0.70 ± 0.09	0.54 ± 0.06	2.51;ns
**Spatial Distribution**						
Center stops (%)	9.3 ± 1.5^2–5^	22.6 ± 4.9^1,5^	55.5 ± 30.8^1^	20.3 ± 1.9^1,5^	41.7 ± 2.8^1,2,4^	3.08; = 0.0277
Center time (%)	2.4 ± 1.1^2,4,5^	19.2 ± 6.1^1,3^	2.4 ± 1.1^2,4,5^	12.8 ± 3.9^1,3,5^	25.5 ± 7.1^1,3,4^	**8.99; <0.0001**
Meander (deg/m)	-1.87 ± 0.31^2^	-0.99 ± 0.36^2^	-1.52 ± 0.19^4,5^	-0.60 ± 0.16^3,5^	-0.46 ± 0.09^3,4^	**7.68; <0.0002**

In a dark arena, the level of activity resembled the highest level that was measured in the illuminated arena, and did not vary significantly with the number of stones (Fig. 1). Thus, activity of spiny mice in a dark arena was steady and high, regardless of the number of stones or their density (Table 2).

**Table 2:** Parameters of locomotion in a dark large arena (2 × 2 m). As shown, level of activity was not affected, whereas the spatiotemporal structure underwent significant changes. Bonferroni adjustment of p-value was calculated by P = 0.05/10 = 0.005. Mean (± SEM) are followed by the results of Tukey HSD test, indicating the numbers of the significantely different test groups (as appeared in the top row).

	Empty (1)	4 Stones (2)	16 Stones (3)	F_18;_p
**Level of activity**				
Traveled distance (m.)	116.51 ± 7.52	110.17 ± 11.58	99.09 ± 10.38	0.91; ns
Speed (m/sec)	0.21 ± 0.02	0.21 ± 0.02	0.20 ± 0.01	0.24; ns
**Temporal Structure**				
Stops	159.1 ± 18.2^3^	185.6 ± 9.4^3^	322.7 ± 32.0^1,2^	**18.67; .0004**
# of trips	40.14 ± 5.53^2,3^	62.57 ± 3.26^1,3^	134.14 ± 13.97^1,2^	**36.68; 0.000001**
Stops/trip	4.04 ± 0.23^2,3^	3.01 ± 0.23^1,3^	2.41 ± 0.04^1,2^	**21.20; 0.00019**
Trip length (m.)	3.08 ± 0.26^2,3^	1.81 ± 0.25^1,3^	0.74 ± 0.04^1,2^	**36.41;0.00000**
Inter-stop distance (m.)	0.77 ± 0.07^2,3^	0.59 ± 0.05^1,3^	0.31 ± 0.01^1,2^	**26.75; <0.00001**
**Spatial Distribution**				
Center stops (%)	25.8 ± 1.3^2,3^	49.8 ± 4.8^1,2^	70.0 ± 1.0^1,2^	**66.09; 0.00000**
Center time (%)	29.3 ± 4.2^2,3^	53.4 ± 6.0^1^	57.7 ± 3.6^1^	**11.58; 0.00058**
Meander	-0.44 ± 0.03^1,2^	-0.33 ± 0.02^1,3^	-0.29 ± 0.05^1,2^	**7.10; 0.0053**

### Temporal organization of locomotor activity

In illuminated arenas, increases in traveled distance were echoed in the number of stops, and it was not possible to distinguish whether the increase in stops was directly linked to increased traveled distance, or whether it was due to the increased number of stones. In the dark arena, however, traveled distance was not different in the three groups (Table 2; Fig. 1), but number of stops increased with number of stones, indicating that stops depended on the number of stones and not on the traveled distance.

**Figure 2:**
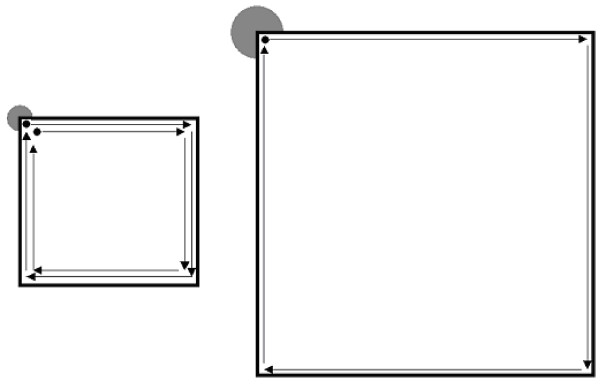
Scaling of interstop distance according to arena size. In the small arena (left illustration) the spiny mouse takes two round trips that start and end at the home base (top left corner), stopping only in the corners of the arena (4 stops/round trip, including the stop at the home base). In the large arena, the spiny mouse takes one trip, stopping only at the corners of the arena (again, 4 stops). Thus, trip length and interstop distance are shorter in the small arena, and the number of trips and overall number of stops are smaller in the large arena. The shorter but more frequent trips in the small arena and longer but fewer trips in a large arena result in the same overall traveled distance and the same number of stops per trip.

The number of trips to the home base increased with the number of stops, which increased with the number of stones. However, the mean number of stops in a trip did not vary in the various groups tested in the illuminated arena (Table 1). As shown in Table 1, in the absence of stones, trip length significantly increased with arena size and spiny mice took fewer but longer trips in the large arena compared with more but shorter trips in the small arena. In addition, inter-stop distance was significantly higher in the large compared with the small illuminated arena. Consequently, the traveled distance was similar in both small and large arenas with same number of stones (Table 1 and Fig. 2).

When the number of trips increased with increase in number of stones, trip-length and inter-stop distance decreased, reflecting the tendency of spiny mice to stop at or near stones. Changes in the number of stops/trip were non-significant (Table 1). Overall, these changes imply that with increase in number of stones, spiny mice set out from the home base to more trips in the arena, but these trips were shorter in distance, had a shorter distance between successive stops, but preserved a relatively invariant number of stops per trip.

A similar trend was evident in the dark arenas, where with increase in number of stones, spiny mice took more trips that were shorter in length and in inter-stop distance. However, the non-significant decrease in the number of stops per trip that was noted in illuminated arenas with increased number of stones, reached statistical significance in the dark. Indeed, the number of stops/trip significantly decreased in 4-stone and in 16-stone arenas (Table 2). Overall, while the level of activity underwent conspicuous changes in illuminated arenas and remained steady in dark arenas, the temporal structure of locomotor behavior underwent similar changes in both illuminated and dark arenas.

### Spatial distribution of locomotor activity and path shape

In empty illuminated arenas, spiny mice spent more than 80% of the time in the corners, the rest of the time mostly along the walls, and as little as 3% of the time in the center. Adding stones changed this pattern and the animals spent 13–26% of the time in the center, as well as stopping more frequently in the center (Table 1). In the dark, however, spiny mice spent 30–60% of the time and 30–70% of their stops in the center, with both percentage of time and stops increasing with increase in number of stones (Table 1).

**Figure 3:**
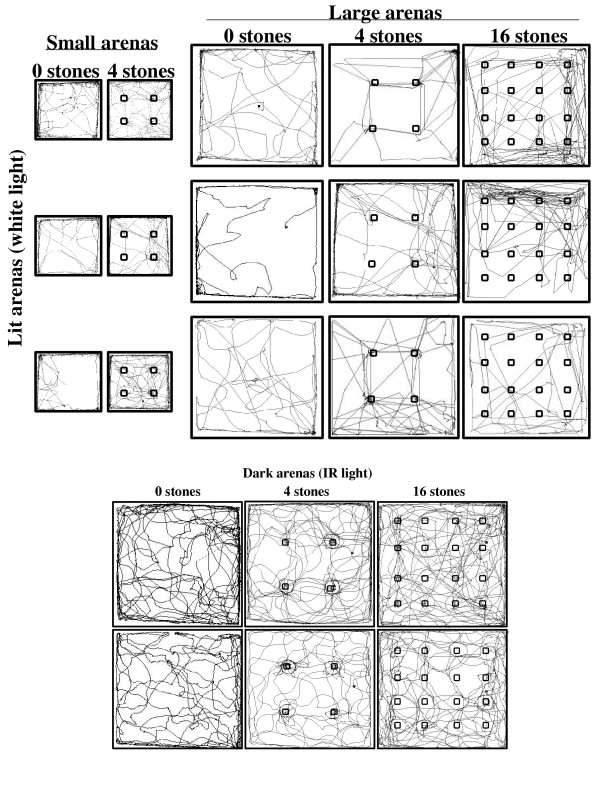
Trajectories of locomotion of exemplary spiny mice in lit arenas (top) and dark arenas (bottom). Each square shows one spiny mouse. As shown, in both small and large empty arenas, either dark or lit, spiny mice moved through the center in a convoluted path, changing frequently the direction of progression. Locomotion in the center increased in the dark or with the number of stones. With stones, trajectories of locomotion comprised more straight segments and fewer changes in direction of progression.

In both small and large empty arenas, either dark or illuminated, spiny mice moved through the center in a convoluted path, changing frequently the direction of progression. When stones were added, trajectories comprised of more straight segments and fewer changes in direction of progression (Fig. 3). This change was reflected in the *Meander *index, which describes the angular change in direction of progression relative to distance moved. As shown, the meander was high without stones, and significantly decreased when stones were added (Tables 2 &3). Changes in the level of activity and its spatio-temporal structure are summarized in Table 3.

**Table 3:** Formal summary of the results shown in Tables 1 and 2.

				Dark *vs*. light			With lights on			With lights off
										
**Level of activity**	Distance traveled and speed			Longer distances			Increase with stone density			Remains high




**Temporal structure**	# of stops and trips			More stops and trips			More stones = more stops and trips			
	Trip length			Shorter trips			More stones = sorter trips			
	Stops/trip			Fewer stops/trip			Did not change			Decreased




**Spatial distribution**	Path shape			Winding (zigzag) paths			More stones = straighter path			
	Time and stops at the perimeter			More time and stops in center			More stones = more time and stops in center			

## Discussion

Spiny mice in the wild inhabit rocky mountains, dwelling in the crevices between and under rocks and boulders. It was therefore assumed that adding stones to an arena would create a complex environment, more resembling their natural habitat. Indeed, in an empty illuminated arena, spiny mice spent extended periods in the corners, traveling mainly along the walls, rarely entering the center of the empty arena where they traveled in a winding path. When stones were placed in the illuminated arena, the animals traveled significantly longer distances, as expected. While it was the density of stones rather than their number that accounted for the increased activity in the illuminated arena, introducing stones into a dark arena did not affect the level of activity, and the distance traveled was high regardless of number of stones or their density. In the following discussion it is proposed that increased activity is due to a sense of security and/or easier navigation provided by the stones whereas the convulted path in empty arena is a defensive strategy or a refelction of a dificulty of navigation in environment without landmarks or shelter (=stones).

Numerous field and laboratory studies found increased activity in nocturnal prey species tested in the dark, compared with their activity when tested under light [2,20]. In the same vein, foraging in rodents was shown to be closely associated with complex areas (shrubs) on bright nights but evenly distributed between sheltered and open areas on dark nights [8,21,22]. This avoidance of open areas probably reflects the finding that rodents are attacked and captured more frequently in the open [23]. It should be noted, however, that this anti-predatory pattern is effective against aerial raptors, but not necessarily against terrestrial predators, as indicated by the increased activity of snakes during dark nights [24], a time when rodents have higher activity. Therefore, the present findings that spiny mice avoid open illuminated spaces while demonstrating a higher level of locomotion in a more complex environment and/or in the dark areas, reinforces previous results on the effect of light level and habitat structure.

The present study demonstrates a change in path shape when locomoting in the center: spiny mice traveled along convoluted trajectories and rarely took straight paths (Fig. 3). These frequent changes in the direction of progression decreased with the increase in number of stones, and were especially conspicuous in empty dark arena, when activity in the center was prevalent. This behavioral pattern is reminiscent of the finding that gerbils' foraging path [25]. A mathematical model [26] suggested that a zigzag trajectory is advantageous when encountering a close or fast predator, whereas a straight trajectory is advantageous in facing a distant and relatively slow predator. Spiny mice may therefore move in a zigzag pattern as a defence against aerial raptors (fast predator) or snakes (close predator). Indeed, when spiny mice were attacked by a barn owl, they continued to locomote fast while frequently changing direction of progression, forming a convoluted path [27].

Another explanation for the changes in path shape is that stones are landmarks, and without them, especially in the dark, spiny mice may have difficulty in navigating [28] and therefore move in a convoluted path. Once landmarks (stones) are available, mice can more easily navigate and travel in straight paths, whereas when stones are absent they travel in the relatively homogenous environment along a winding path. A reminiscent mechanism was described in desert ants (*Cataglyphis fortis*) that return to nest directly but not necessarily in a straight path, presumably turning as frequently to the right as they do to the left, to reduce overall directional bias [29]. A survey of the mechanisms that may underlie intermittent progression suggests that pauses increase the capacity of sensory systems to detect relevant stimuli, and may involve perceptual processes such as velocity blur, relative motion detection, foveation, attention and interference between sensory systems [30]. When stones (=landmarks) are present, stops are frequent and spatial information can be collected during stops, alowing traveling along straight trajectories, whereas the lack of such spatial information processing may result in a winding path, as seen in empty and/or dark arena.

Light condition affected the spatial distribution of locomotor activity: while spiny mice remained most of the time along the walls of empty illuminated arenas, they increased the center time by 5–10 folds in complex environments. That the animals spent more time close to the walls in the empty illuminated arena compared with dark arena or complex arenas is unsurprising, probably linked to thigmotaxis, as shown in other rodent species (e.g., [20,31-33]). In the dark arena, however, center time and stops in the center were distinctly higher than in the illuminated arena, comprising 25%–70% of activity. This further supports the assumption that spiny mice move more in the center when afforded shelter by darkness and/or by the physical structure of the environment.

Stopping may also have an anti-predatory role [34] since owls usually attack moving prey, after being stimulated by its movement [35-37]. In consequence, a common defensive strategy in prey species is to freeze and remain immobile in the face of life threat, in order to eliminate the auditory and visual cues that predators use in pinpointing prey [38]. In following the above discussion on a possible defensive significance of convoluted paths, it is possible that complex environment in the dark does not provide the same sense of security than it does in an illuminated arena. This could be a result of snake activity, which is higher in dark and complex habitats but lower in the open [22,24,31]. It should be noted, however, that the above explanations are not mutually exclusive, and stopping may have a synergistic role in orientation, physiological recovery, and anti-predatory defense [34].

The present results in spiny mice are thus consistent with previous similar results in rats and voles [15,16]. in that they indicate that the animals preserve activity level and temporal structure under changing arena size. This observation may be a general property of rodents' open field behavior, which is gained by scaling interstop distance and number of trips to the home base. When environmental complexity was increased by adding stones, the number of trips increased while their length decreased. Therefore, the higher level of locomotor activity in complex environments was the result of more frequent but shorter trips and not of longer trips. This was obvious in the dark arena, where level of activity was high regardless of environment complexity, while the number of trips increased and their length decreased with increase in space complexity. These differences in the structure of trips may serve as a search-image parameter in other studies in spiny mice. For example, it is expected that foraging (e.g. traveling to food patches) will be longer in distance and less frequent in illuminated or exposed environments, but shorter and more frequent in a dark or sheltered area [39]. Long trips in the open are more risky, however, and spiny mice therefore need to undertake measures that reduce this risk. One possible way of reducing risk may be achieved by changing the distribution of activity and path shape, as described above.

## Conclusions

The present results follow previous studies that demonstrated lower activity in illuminated and open areas compared with dark and complex areas. Observations on the behavior of spiny mice under these conditions revealed changes in the number and length of trips, in stopping frequency, and in path shape. Altogether, these changes reflect a flexible adaptation of locomotor activity to environmental conditions in a way that may be interpreted as aimed at efficient navigation, preserving the temporal structure of behavior, and reducion of predatory risk.

## Methods

### Study animals

The common spiny mouse (*Acomys cahirinus*) weighs 38–44 g and is 11 cm long, plus a 10-cm tail [40]. Spiny mice are an exceptional genus among murid rodents (Muridae) in being precocial and not having a nest. They differ from rats and mice in many aspects (see [41] for review); noteworthy are differences in depth perception [42], distance perception [43], exploration [44] and excitability [45]. We obtained 71 adult spiny mice bred in captive colonies at the research zoo of Tel-Aviv University. Fifty spiny mice were divided into five groups (n = 10; five males and five females per group); these groups were tested in a illuminated arena. The other 21 spiny mice were divided into three groups (n = 7; 3–4 males and 3–4 females per group); these groups were tested in a dark arena. The larger group size in light tests was due to the greater behavioral variability in light compared with dark tests. Several weeks before testing, the animals were housed in groups of 5–10, in metal cages measuring 40 cm × 70 cm and 25 cm, located outdoors in the zoo yard under natural (uncontrolled) temperature and light conditions. Overturned ceramic pots and wooden boxes were placed in each cage to provide shelter. Seeds, diced fresh vegetables, and live fly larvae were provided ad lib. Based on years of experience in maintaining colonies of spiny mice in our zoo, provision of water is unnecessary when sufficient fresh vegetables are provided.

### Apparatus

A test arena was constructed by enclosing a tiled floor with plywood planks (50 cm high). Two arena sizes were used: 1 × 1 m and 2 × 2 m. Stones (tiles), 12 cm long, 12 cm wide and 6 cm high, were placed in the arena (details below). The arena was located inside an air-conditioned room (24°C), and could be illuminated by one of the following light-sources: (1) two 300 W light bulbs directed to the white ceiling in order to provide diffused illumination of the arena (Light tests); (2) two infrared lights (*Tracksys*, IR LED Illuminator; UK) that emit light in a range invisible to rodents (Dark tests; light level was 0.0425 Lux as measured with Profisix Sbc, *Gossen*). The video signal was recorded on a VCR (JVC HR-J737).

### Procedure

*C*ages with spiny mice were brought to a room adjacent to the testing room 10 h before testing. For testing, a spiny mouse was removed in random order from the cage to a jar, and gently released from the jar into the center of the arena. Each spiny mouse was tested only once for 10 minutes, being randomly assigned to one of the above arenas. At the end of testing, animals were returned to the population at the research zoo. The first five groups of spiny mice (n = 10/group) were all tested in illuminated arenas under the following conditions: (1) small empty arena (no stones); (2) small arena with 4 stones; (3) large empty arena; (4) large arena with 4 stones; and (5) large arena with 16 stones (see Fig. 1). It should be noted that a setting of a small arena with 16 stones would have virtually prevent locomotion, and was therefore excluded. Three additional groups (n = 7/group) were tested in a large (2 × 2 m) dark arena with: i) no stones; ii) four stones; and iii) 16 stones, in order to compare the behavior in these three arenas with the behavior in the respective illuminated arenas.

### Behavioral analysis

A tracking system (*Ethovision *by Noldus, NL) was used for data acquisition. The tracking system was set to score the spiny mouse as "not moving" (=stopping) when its center of gravity moved at a speed lower than 2 cm/sec, or as "moving" when the speed exceeded this limit during tracking at a rate of 25 frames/sec. Each arena was divided into four zone types. These were ***corners ***– a 20 × 20 cm square at each of the four corners of the arena; ***walls ***– a 20 cm strip along the walls between the corner zones; ***stones ***– a 20 × 20 cm square centered with each stone; ***center***, the remaining area that comprised the spaces between the stones and away from the walls and the corners of the arena.

Based on our past studies, the parameters acquired from Ethovision were classified to represent three perspectives: i) level of activity, ii) temporal organization of locomotion, and iii) spatial distribution of locomotion. The parameters that were measured are described in Table 4. Briefly, the level of activity refers to the amount of activity regardless of temporal structure of spatial distribution. For example, the metric distance traveled was measured regardless of whether it comprised intermittent or continuous locomotion, or whether it was along the perimeter or in the center of the arena. The temporal structure refers to the order of bouts of locomotion and stopping periods. Parameters on temporal organization were derived in past studies, showing that locomotor behavior is organized in relation to a home base; a place where a rodent spends the longest cumulative non-locomoting periods [11,12,16]. From the home base the rodent takes round trips in the environment. The spatial distribution was aimed at distinguishing where activity had occurred. For example, the same amount of activity with the same temporal structure could be executed along the perimeter of the arena, or only in its center. Alternatively, a spiny mouse could travel in a straight or a winding trajectory. For these, the representation of the spatial distribution of activity was required.

**Table 4:** Parameters of locomotion that were measured for each spiny mouse.

Behavior	Description
**Level of activity**	
Distance traveled	Overall distance (m.) that a spiny mouse traveled during the 10-min observation.
Traveling speed	Overall traveled distance divided by the duration of locomoting periods (m/sec).
**Temporal organization**	
Number of stops	Incidence of "non-locomoting" intervals (stops), bounded by locomotion.
Number of trips	Trips are intervals between consecutive stops at the home base, which is the place with the highest rank among zones according to the accumulated "non-locomoting" intervals. Thus, a trip comprised progression out from home base through consecutive stops in the arena, until returning to the home base.
Stops/trip	Number of stops taken between two successive visits to the home base (= total number of stops divided by the total number of trips).
Trip length	Metric distance traveled in a round-trip to the home base (total distance divided by the total number of trips).
Inter-stops distance	The metric distance traveled between two consecutive stops (or, distance traveled in a "locomoting" interval = distance divided by number of stops).
**Spatial distribution**	
Time spent along the perimeter (%)	Calculated as percentage of the total time, in order to show how long spiny mice stayed at the vicinity of the walls of the arena, compared with the time spent in the center of the arena or near/on the stones.
Stops along the perimeter (%)	Calculated as percentage of the total stops, in order to show how many stops took place along the vicinity of the walls of the arena, compared with stopping in the center of the arena or at/on the stones.
Meander	The rate of change in direction of progression relative to the distance traveled, calculated automatically by Ethovision for each two successive time points by dividing the turn angle by the distance. Mean meander of each spiny mouse was used to calculate the mean of each group. + indicated a clockwise change in direction of progression, whereas - indicated a counterclockwise change. Thus, lower absolute (+ or -) meander values characterize locomotion along relatively straight trajectories, and higher meander absolute values describe circular or winding trajectories. It should be noted that meander is sensitive to tracking rate, animal size, and arena size. Therefore, meander may be compared only for the same animal size, same resolution, and same arena size.

### Statistics

One way analysis of variance (ANOVA) was applied. Some of the data may not be strictly independent – i.e. trip length is the division of traveled distance by the number of trips, etc. – therefore, a Bonferroni correction was applied to set alpha level to 0.005 (0.05 divided by the 10 parameters that were used). Data calculated as proportions were transformed to the arcsine of the square-root-transformed raw data.
